# Non-thermal ablation and drug delivery by electroporation: expanding current indications beyond cancer and cardiac treatment

**DOI:** 10.2478/raon-2026-0040

**Published:** 2026-08-12

**Authors:** Damijan Miklavcic, Helena Cindric, Rafael V Davalos, Frederic Deschamps, Julie Gehl, Bor Kos, Peter Kramar, Dimitrij Kuhelj, Martijn R Meijerink, Declan Soden, Miha Stabuc, Gregor Sersa

**Affiliations:** 1 University of Ljubljana, Faculty of Electrical Engineering, Ljubljana, Slovenia; 2 Department of Biomedical Engineering, Georgia Tech – Emory University, Atlanta, United States; 3 Department of Anaesthesia, Surgery and Interventional Procedures (DACI), Gustave Roussy, Villejuif, France; 4 Centre for Experimental Drug and Gene Electrotransfer (C*EDGE), Department of Clinical Oncology and Palliative Care, Zealand University Hospital, Roskilde, Denmark; 5 Department of Clinical Medicine, Faculty of Health and Medical Sciences, University of Copenhagen, Copenhagen, Denmark; 6 SIQ | Slovenian Institute of Quality and Metrology, Ljubljana, Slovenija; 7 Clinical Institute of Radiology, University Medical Centre Ljubljana, Ljubljana, Slovenia; 8 University of Ljubljana, Medical Faculty, Ljubljana, Slovenia; 9 University of Novo mesto, Faculty of Health Sciences, Novo mesto, Slovenia; 10 Department of Radiology and Nuclear Medicine, Amsterdam University Medical Centre, Amsterdam, Netherlands; 11 Mirai Medical, Galway, Ireland; 12 Department of Experimental Oncology, Institute of Oncology Ljubljana, Ljubljana, Slovenia

**Keywords:** electroporation, pulsed field ablation, electrochemotherapy, irreversible electroporation, gene electrotransfer

## Abstract

**Background:**

Electroporation-Based Treatments and Therapies (EBTTs) – electrochemotherapy, irreversible electroporation, pulsed field ablation, and gene electro-transfer – use short, high-voltage electric pulses to permeabilize cell membranes, enabling non-thermal tissue ablation and enhanced local drug delivery. Although EBTTs have been used clinically for over three decades, primarily in oncology, their adoption remains limited compared to thermal ablation modalities. In contrast, cardiac pulsed field ablation (PFA) has seen rapid adoption since the first FDA device approval in December 2023.

**Conclusions:**

This expert opinion paper reviews the current clinical landscape of EBTTs in oncology and cardiac arrhythmia treatments, and focuses on emerging non-oncological and non-cardiac applications, including endoscopic pulsed electric field therapy for type 2 diabetes, bronchial rheoplasty for chronic bronchitis, bleomycin electrosclerotherapy for vascular malformations, nanosecond PFA for benign thyroid nodules, and electroporation-mediated gene delivery – and identifies key barriers to wider adoption: insufficient randomized evidence, lack of validated treatment planning tools, fragmented reimbursement, and limited standardization. We outline measures to facilitate clinical translation.

## Introduction

Electroporation is a biophysical phenomenon in which the application of short, high-voltage electric pulses disrupts the lipid bilayer of the cell membrane. Depending on pulse parameters, namely amplitude, duration, number, and repetition rate, the effect is either reversible or irreversible.^1^ This allows for a family of clinically distinct treatment modalities. In electrochemotherapy (ECT), reversible electroporation potentiates the cytotoxic effect of chemotherapeutic agents such as bleomycin and cisplatin, increasing their intracellular uptake by orders of magnitude.^2,3^ In a similar manner, gene electro-transfer (GET) facilitates intracellular delivery of therapeutic nucleic acids.^4,5^ In irreversible electroporation (IRE) ablation, the cell membrane is disrupted, causing irreversible cell damage through a predominantly non-thermal mechanism, while preserving the extracellular matrix, vascular scaffolding, and critical anatomical structures.^6,7^ The same physical principle, when applied as pulsed field ablation (PFA) via intracardiac catheters, now constitutes the fastest-growing treatment modality in cardiac electrophysiology for the treatment of atrial and ventricular fibrillation.^8,9^

Electroporation Based Treatments and Therapies (EBTTs) offer several inherent advantages over conventional thermal ablation modalities such as radiofrequency ablation (RFA), microwave ablation (MWA), and cryoablation (Cryo). Because cell death is driven primarily by electric field mediated membrane disruption rather than heating, EBTTs can be used in proximity to heat-sensitive critical structures, including bile ducts, bowel walls, and major nerves, without the thermal collateral damage that limits conventional ablation in these anatomical locations.^10,11^ EBTTs are also not subject to the heat-sink effect that compromises thermal ablation efficacy near large vessels, and they produce a vascular lock effect that reduces intraprocedural bleeding.^12,13^ Emerging evidence further suggests that electroporation-based ablation releases tumour-associated antigens in their native, nondenatured state, eliciting a more robust pro-immunogenic response than thermal modalities and thereby opening a therapeutic window for combination with immune checkpoint inhibitors.^14-17^

Despite these advantages and a clinical track record spanning more than 30 years, the first ECT clinical trial was published in 1991^18^, EBTTs remain underutilised in clinical practice. ECT remains primarily applied to cutaneous and subcutaneous tumours, and both ECT and IRE ablation are expanding into interventional oncology for deep-seated tumours; however, both remain niche techniques compared with thermal ablation modalities, which constitute the current standard of care for most tumour indications. In striking contrast, cardiac PFA has experienced explosive growth: following the first FDA clearance of Medtronic’s (Minneapolis, MN, USA) PulseSelect system in December 2023 and Boston Scientific’s (Marlborough, MA, USA) Farapulse system in February 2024, approximately one million patients have been treated worldwide within two years, fuelled by substantial research and development investment and corporate acquisitions. The cardiac PFA experience demonstrates both the translational potential of electroporation and the conditions required for rapid clinical adoption: a large, homogeneous patient population, compelling randomised evidence, sustained industrial investment, and a clear regulatory pathway.

This expert opinion paper examines whether and how these treatments can be adapted for other non-cardiac and non-oncological applications of electroporation. We review the current clinical landscape of EBTTs, explore emerging indications beyond oncology and cardiology, analyse the barriers to wider clinical adoption, and propose concrete steps to accelerate clinical translation.

## Current clinical landscape of electroporation-based treatments

### Electrochemotherapy

Electrochemotherapy (ECT) has the longest clinical history among EBTTs. Following the pivotal 1991 clinical trial by Mir *et al*., the 2006 Standard Operating Procedure (SOP) and its 2018 update^19,20^ and the NICE (National Institute for Health and Care Excellence) approval of ECT for skin metastases in the UK in 2013 (IPG446), the treatment is now routinely performed in Europe. The Insp-ECT (International Network for Sharing Practices on Electrochemotherapy) registry (https://www.insp-ect.org/), encompassing more than 40 treatment centres and over 1,000 analysed patients, treated with Cliniporator devices (IGEA, S.p.A., Carpi, Italy), has demonstrated overall response rates exceeding 80% across diverse tumour histologies, with complete response rates of approximately 71%.^21,22^ Mirai Medical’s ePORE® platform has also been applied to cutaneous malignancies through the CUTIS probe. In a case series (25 patients), high-frequency electroporation with the ePORE® generator and CUTIS probe, combined with bleomycin, achieved a 91.3% overall lesion response at 12 weeks across 97 lesions of five histological subtypes, with good tolerability under local anaesthesia.^23^

Beyond cutaneous disease, ECT is increasingly applied to deep-seated targets including liver, prostate, bone, colorectal, vulvar, and brain tumours.^21,24–31^ A particularly compelling application is ECT for metastatic epidural spinal cord compression, a devastating condition with few therapeutic options beyond palliative radiotherapy. The largest published series of ECT for metastatic epidural spinal cord compression, reported by Deschamps *et al*., included 40 consecutive patients who had failed radiotherapy; 46% achieved complete MRI response and the median pain score fell from 7/10 to 1/10 at one month.^32,33^ These results represent a promising change in quality of life for a patient population with previously no treatment options.

ECT has also been delivered endoscopically for gastrointestinal indications. The EndoVE® platform (Mirai Medical, Galway, Ireland) has accumulated the most extensive clinical experience in endoscopic, intraluminal gastrointestinal electroporation. Following the first-in-human colorectal study^34^, independent groups have since reported endoscopic calcium electroporation and ECT with this platform across colorectal, gastric and oesophageal cancer, principally for palliation of bleeding and obstructive symptoms in frail patients unfit for surgery, with a consistent safety profile and high rates of symptomatic response.^35-41^ A standardised implementation protocol for an endoscopic electroporation service in colorectal cancer has also been published.^36^

A few CE-marked clinical electroporators are currently approved for ECT in Europe. Cliniporator (IGEA S.p.A., Carpi, Italy), which received CE marking for ECT in 2008, remains the most widely used system and is the device employed across the Insp-ECT registry. A higher-output variant, the Cliniporator VITAE, which extended ECT to deepseated and larger tumours such as those of the liver, bone, and spine, received a CE mark in 2011. The SENNEX System (BionMed Technologies, Saarbrücken, Germany) is also CE-marked for ECT of cutaneous and subcutaneous tumours.^42^ Mirai Medical (Galway, Ireland) has developed the ePORE® high-frequency biphasic generator and the EndoVE® endoscopic electrode^34^, both CE-approved for ECT and calcium electroporation in the EU, UK and Australia, enabling endoscopic delivery of ECT for gastrointestinal indications.

### Irreversible electroporation in interventional oncology

Irreversible electroporation (IRE) was first suggested as a non-thermal ablation modality in 2005^6^, demonstrated in vivo in 2007^43^ and finally introduced to the market by AngioDynamics (Latham, NY, USA), whose NanoKnifeÒ electroporator received FDA clearance for soft tissue ablation in 2008. IRE has been most extensively studied in the liver, pancreas, and prostate, organs where proximity to critical structures such as the hepatic hilum, bile ducts, and neurovascular bundles, or the heat-sink effect of adjacent vasculature, limits the effectiveness of thermal ablation techniques.^10,44-49^ Retrospective and prospective studies have demonstrated technical feasibility and acceptable safety profiles of the procedure.

Clinical interest in IRE for prostate cancer is increasing, as preservation of the neurovascular bundles is essential for maintaining continence and potency. The PRESERVE study (NCT04972097), a recent clinical trial, demonstrated the safety and effectiveness of IRE for treatment of prostate. Most recently, Angiodynamics’ NanoKnifeÒ system received FDA clearance in December 2024 for ablation of prostate tissue in intermediate-risk prostate cancer, further expanding the approved indications for IRE.

There is also growing interest in early-stage non-small cell lung cancer, where the proximity to major airways and vessels limits the use of thermal approaches. Recent regulatory milestones reflect this momentum: Galvanize Therapeutics’ (Redwood City, CA, USA) Aliya system received FDA clearance in 2022, and the INUMI Flex endoscopic needle in 2024, both supporting soft tissue ablation, including endobronchial treatment of early-stage non-small cell lung cancer. Beyond lung, regulatory clearances continue to expand into other soft tissues; Pulse Biosciences’ (Hayward, CA, USA) CellFX nsPFA technology, which received FDA 510(k) clearance in March 2024 for ablation of soft tissue in percutaneous and intraoperative surgical procedures.

### Cardiac pulsed field ablation

IRE has also gained momentum with its evolution to Pulsed Field Ablation (PFA), a non-thermal modality for intracardiac catheter-based ablation, unlocking new possibilities for the treatment of cardiac arrhythmias.^50-52^ In cardiology, PFA has achieved rapid regulatory approval and clinical adoption for the treatment of atrial fibrillation. Medtronic’s (Minneapolis, MN, USA) PulseSelect system was the first PFA device approved by the FDA for the treatment of atrial fibrillation in December 2023. This was followed in 2024 by FDA approvals for Boston Scientific’s (Marlborough, MA, USA) Farapulse, Affera/Medtronic’s Sphere 9, and Johnson & Johnson’s (New Brunswick, NJ, USA) VARIPULSE, and in 2025 by approvals for Kardium’s (Burnaby, BC, Canada) Globe System and Abbott’s (Abbott Park, IL, USA) Volt PFA System.

Substantial investment in research and development has supported large-scale randomised trials such as ADVENT, CHAMPION, and BEAT PAROXAF^53-55^, along with prospective pivotal studies such as PULSED AF and ADVANTAGE AF^51,56^, which have validated the technology platform.^57,58^ PFA is now being evaluated across a broader range of cardiac arrhythmias. In ventricular arrhythmias, PFA’s ability to potentially penetrate fibrotic and fatty myocardium makes it mechanistically attractive for ventricular tachycardia ablation. Early multicentre registry data (126 patients) reported approximately 78% freedom from recurrence for premature ventricular contractions and 70% for ventricular tachycardia at a mean follow-up of 5.6 ± 3.7 months.^58^ A subsequent single-centre US registry (59 patients) using the Sphere-9 catheter reported acute VT non-inducibility in 78% of patients and complete suppression in all PVC cases, with 6-month VT-free survival of 69.8%.^59^ For supraventricular tachycardias, acute procedural success rates exceeding 99% have been reported in early multicentre studies.^58^

The trajectory of cardiac PFA offers both inspiration and a sobering reality check for non-cardiac electroporation. The heart is an anatomically consistent target across patients, the global atrial fibrillation population is vast, and the existing catheter ablation infrastructure could be readily adapted; allowing a single catheter and technique to be applied across the population. Oncological targets, by contrast, span every tumour histology and anatomical site, each demanding different electrodes, specialists, and procedural approaches. This heterogeneity makes a single pivotal trial for oncological EBTTs impractical and calls for alternative trial design. It also carries a regulatory cost: each indication must be evaluated separately, so the evidentiary burden scales with the number of target conditions rather than being shared across one large population, as in atrial fibrillation.

## Emerging applications beyond oncology and cardiology

The success of cardiac PFA has demonstrated that electroporation is a viable treatment platform for non-oncological conditions. Several new clinical applications are now at various stages of development, each leveraging the unique properties of pulsed electric fields, cell selectivity ablation, preservation of tissue architecture, and enhanced drug delivery for conditions that were not traditionally considered targets for electroporation.

This section examines the emerging applications of electroporation beyond oncology and cardiology. The variety of terms in use (e.g., pulsed electric field therapy, pulsed field ablation, (ir)reversible electroporation, nanosecond pulsed field ablation), reflect the clinical discipline and context of origin rather than mechanistic distinction; all share electroporation of the cell membrane as their underlying mode of action.

### Endoscopic pulsed electric field therapy for type 2 diabetes

**Figure 1.**
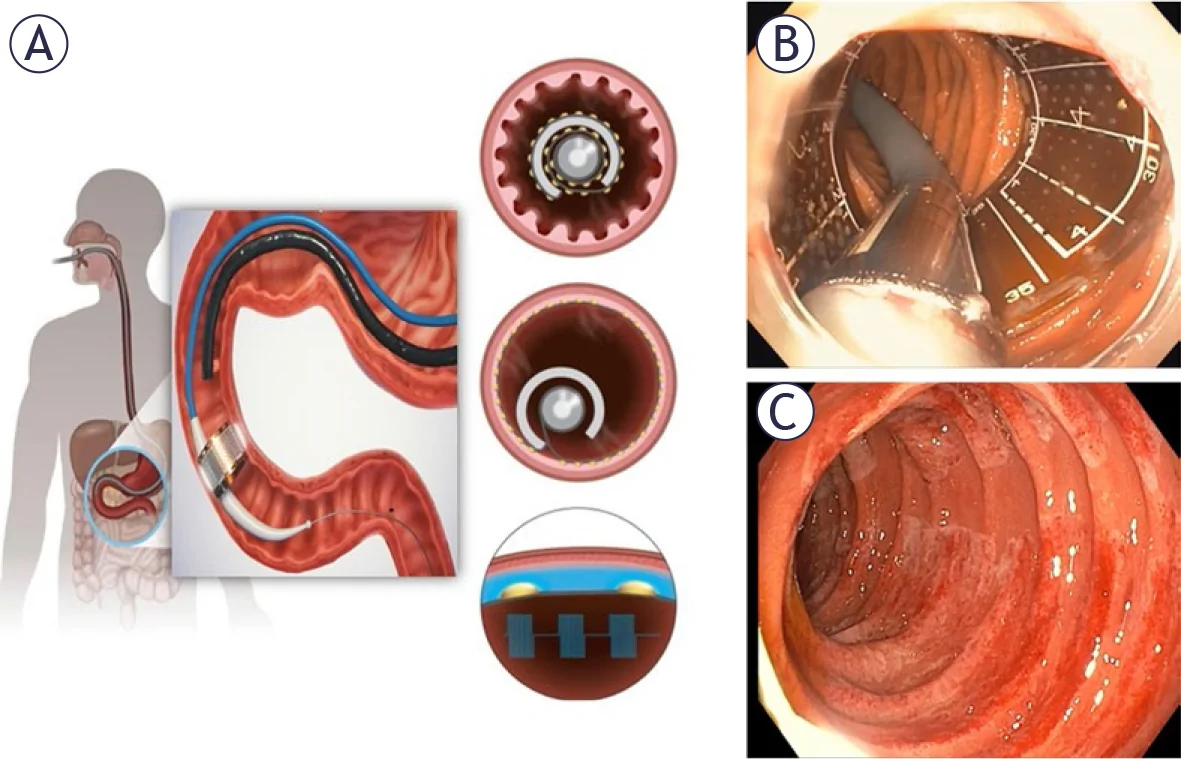
Recellularization via electroporation therapy (ReCET) is a novel endoscopic procedure that uses an endoscope-mounted electrode to deliver pulsed electric fields to the duodenal mucosa and submucosa. **(A)** A schematic of the positioning of the flexible electrode and its unfolding and expansion within the duodenum. **(B)** Intraprocedural endoscopic view of the expanded electrode within the duodenum. **(C)** A post-treatment endoscopic view of the resulting circumferential, nonthermal treatment effect. Figure is reproduced from.^62^

Type 2 diabetes mellitus (T2D) affects more than 400 million people worldwide and is responsible for approximately two million deaths annually.^60^ The duodenal mucosa plays a central role in metabolic regulation. Structural and functional abnormalities of the duodenal mucosa are increasingly recognised as contributors to insulin resistance and dysglycaemia, suggesting that endoscopic restoration of the mucosal layer may complement existing pharmacotherapy. Recellularization via electroporation therapy (ReCET) is a novel endoscopic procedure that uses an endoscope-mounted electrode, the Endogenex System (Endogenex Inc., Plymouth, MN, USA), to deliver pulsed electric fields to the duodenal mucosa and submucosa, inducing controlled cellular apoptosis and subsequent re-epithelialisation aimed at restoring normal metabolic signalling (Figure 1).^60,61^

Two clinical studies have generated encouraging early feasibility data. In the EMINENT^60^ first-in-human study (n = 14), insulin-dependent T2D patients underwent a single ReCET procedure. At 12 months, 86% of patients remained off exogenous insulin with HbA1c maintained below 7.5%, and this effect was durable through 24 months of follow-up. No serious adverse events were observed in the studies.

Separately, the REGENT-1 programme^61^ (two parallel multicentre studies in Australia and the United States, n = 71) evaluated ReCET across multiple energy doses in patients on non-insulin glucose-lowering medications. No device-or procedure-related serious adverse events occurred; in the highest-dose group using a second-generation endoscope electrode, mean HbA1c decreased from 8.8% to 7.2% at 24 weeks, with a clear dose-response relationship observed across treatment groups.

These are single-arm studies, and the relative contributions of ReCET and concomitant pharmacotherapy cannot yet be separated. Two randomised, sham-controlled trials are now underway, the pivotal ReCET study (NCT06267391) and the EMINENT-2 trial in Europe (NCT05984238), which aims to provide the comparative evidence needed for regulatory approval. The Endogenex System has received FDA Breakthrough Device Designation, and the company has secured Series C financing to support its pivotal study and regulatory path to FDA approval.

### Bronchial rheoplasty for chronic bronchitis

Chronic bronchitis, a phenotype of Chronic Obstructive Pulmonary Disease (COPD), is characterised by goblet cell hyperplasia and mucus hypersecretion, leading to persistent cough, sputum production, impaired quality of life and increased exacerbation risk. Currently available pharmacotherapies do not adequately target the underlying mucus hypersecretion, leaving many patients symptomatic despite optimal medical therapy.

**FIGURE 2.**
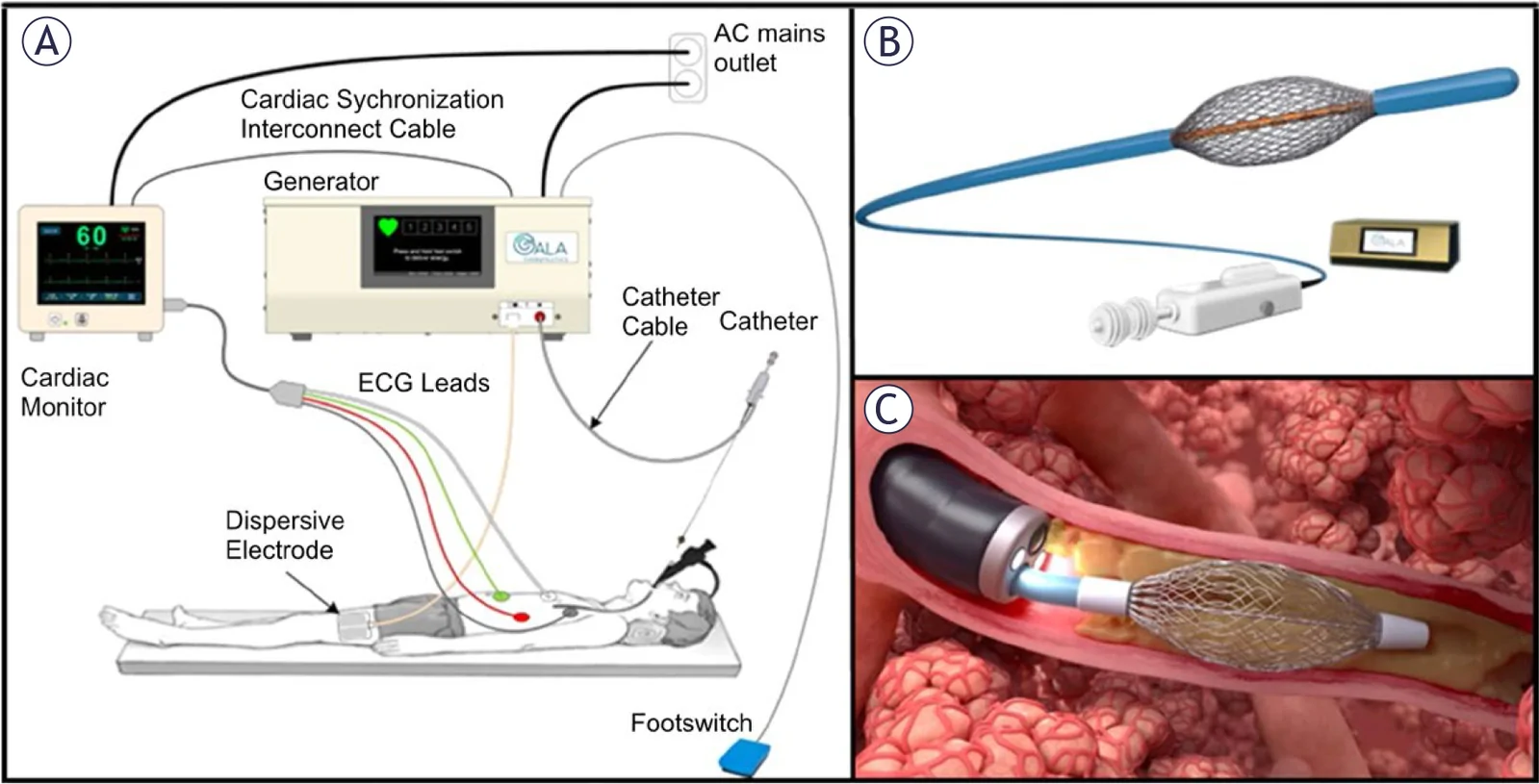
**(A)** The RheOx system (Galvanize Therapeutics, Redwood City, CA, USA) for the treatment of the symptoms of chronic bronchitis consists of a proprietary electrosurgical generator and **(B)** a single use endoscope with an expandable basket electrode. **(C)** The minimally invasive bronchoscopic therapy delivers short bursts of Pulsed Electric Field (PEF) energy to induce non-thermal ablation of the abnormal mucus-producing cells in the airways, leaving the extracellular matrix intact so that the epithelium may rapidly regenerate. Figure is reproduced from.^66^

Bronchial rheoplasty is a bronchoscopic procedure that uses the RheOx system (Galvanize Therapeutics, Redwood City, CA, USA) to deliver non-thermal pulsed electric fields (PEF) to the airway epithelium and submucosa via an endobronchial endoscope electrode (Figure 2). PEF leaves the extracellular matrix and collagenous structures intact, thereby allowing re-epithelialisation with a normalised distribution of mucus-producing cells.^63-65^

A US multicentre feasibility study (n = 21, 6 centres) with 2-year follow-up reported statistically significant improvements in COPD Assessment Test (CAT) and St George’s Respiratory Questionnaire (SGRQ) scores, with no procedure-related serious adverse events.^64^ Mean changes from baseline in CAT score were 9.0 ± 6.7 at 12 months and 5.6 ± 7.1 at 24 months, and in SGRQ 16.6 ± 13.2 and 11.8 ± 19.2, respectively. Both scores exceeded twice the minimal clinically important difference at 24 months. There was also a 34% reduction in moderate and a 64% reduction in severe COPD exacerbation rates compared with the year prior to treatment.

A real-world European registry (n = 54, 106 procedures across 8 centres in Austria and Germany) confirmed these findings^65^, with CAT score improvements of 5.5 ± 8.0 at 6 months and 4.2 ± 7.4 at 12 months, and SGRQ improvements of 9.5 ± 17.5 and 12.4 ± 16.3, respectively. Notably, patients with a CB-dominant phenotype demonstrated substantially higher responder rates than those with a mixed emphysema/CB phenotype (81.6% vs. 36.3% at 6 months), suggesting that patient selection may be important for optimising outcomes.

The RheOx system holds CE certification in the European Union (since 2019) and has been granted FDA Breakthrough Device designation in the United States, where it remains investigational. The pivotal RheSolve trial (NCT04677465), a prospective, randomised, double-blind, sham-controlled, multicentre study enrolling 270 patients at up to 50 centres in the US, Canada and Europe is currently underway to confirm these findings and support a future FDA approval application.

### Bleomycin electrosclerotherapy for vascular malformations

Vascular malformations are congenital anomalies arising from defective vascular morphogenesis and affect approximately 1.5% of the general population.^67^ Slow-flow venous and lymphatic malformations are the most common subgroup, typically presenting during childhood with pain, swelling, functional impairment, and recurrent infections.^68^ Currently available treatments (embolization, bleomycin sclerotherapy, laser therapy, and surgery) are not reliably curative for larger or infiltrative lesions, and recurrence is common.^67-69^

**FIGURE 3.**
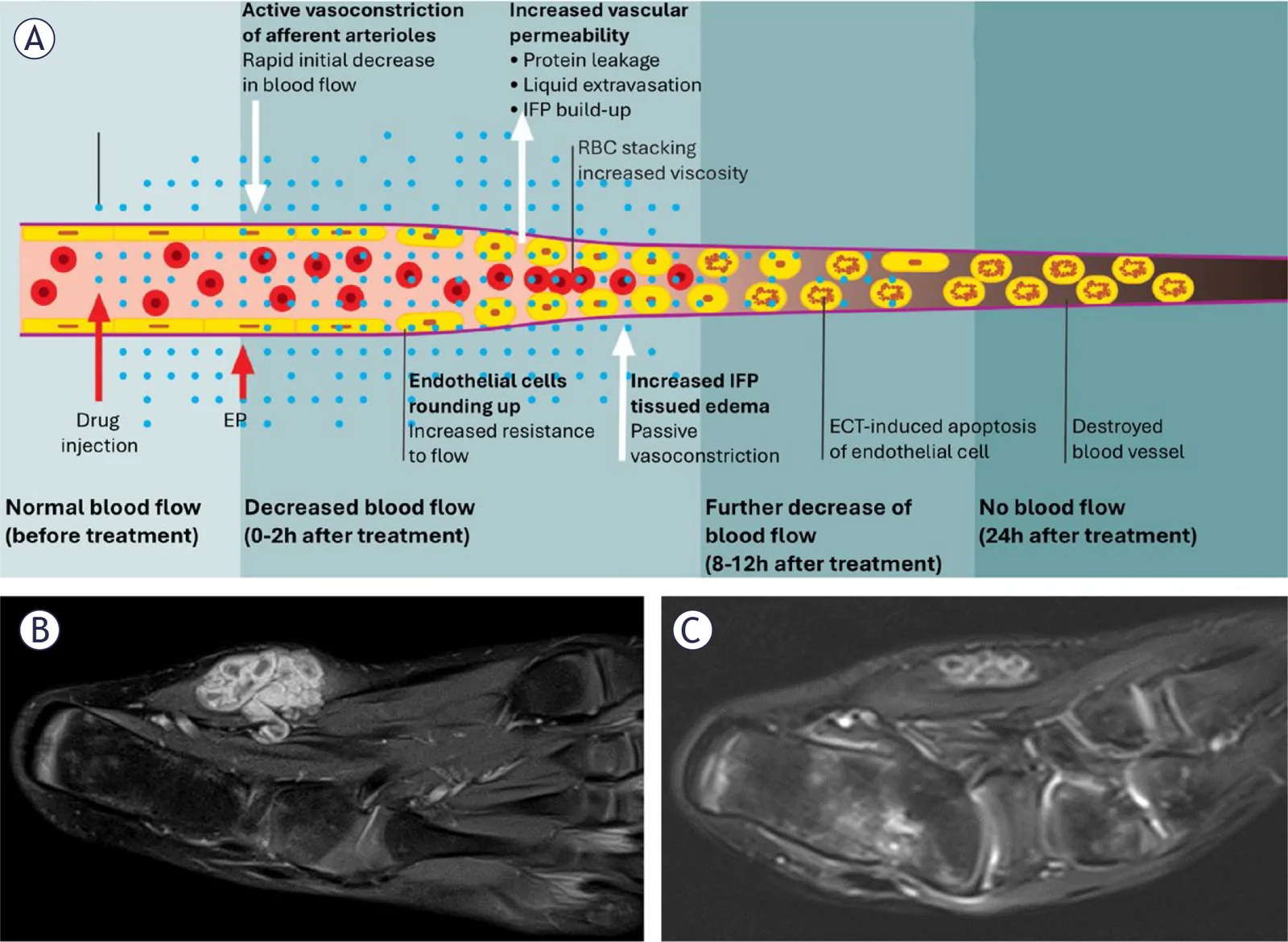
Bleomycin ElectroScleroTherapy (BEST) combines intralesional bleomycin with reversible electroporation, exploiting the same vascular-disrupting mechanism described for electrochemotherapy (ECT). **(A)** An illustration of blood-flow changes induced by electrochemotherapy (ECT) at the level of a microcirculatory vessel; from normal flow before treatment to vascular destruction by 24 h. Adapted from.^12^ An example of BEST treatment of a vascular malformation in the foot: **(B)** T2-weighted MRI of a vascular malformation in the foot before BEST treatment, showing the hyperintense malformation. **(C)** MRI of the same region 3 months after BEST, demonstrating a 75% volume reduction.

Bleomycin ElectroScleroTherapy (BEST) combines intralesional bleomycin with reversible electroporation, exploiting the vascular disrupting mechanism as identified and described in ECT (illustrated in Figure 3 A).^12,70^ Electric pulses transiently permeabilise endothelial cells, substantially increasing intracellular bleomycin uptake; the resulting mitotic cell death preferentially affects proliferating endothelium, a feature shared by tumour vasculature and the dysplastic endothelium of vascular malformations.^67,69,71^

An early cohort of therapy-resistant venous malformations (n = 17, who had previously undergone at least two unsuccessful invasive treatments) reported a median 86% MRI-derived volume reduction after a single BEST session in most cases.^72^ Figure 3 B-C) shows an example of a vascular malformation in the foot that exhibited an approximately 75% volume reduction after BEST. The largest published cohort to date (n = 233 patients, 325 procedures) confirmed positive subjective outcomes in mobility, aesthetics, and pain in most patients. Importantly, children and adolescents performed significantly better than adults across all patient-reported parameters.^73^

Loeser *et al*.^74^ reported BEST outcomes specifically in lymphatic malformations (12 patients, 21 procedures) with mean volume reduction of 54.8% and symptom improvement achieved in all patients after a mean follow-up of 8.36 months. Haehl *et al*.^68^ in the largest dedicated paediatric cohort (45 children, 68 procedures), demonstrated significant improvement in physician-rated symptom severity, with no patient remaining in the severe grade after treatment.

The most common side effect is skin hyperpigmentation (approximately 69%), which frequently fades over time; serious complications, such as skin necrosis, nerve injury, and haemorrhage, are uncommon but not negligible, the reported major complication rate in the largest cohort was 8.9%.^73^ The first consensus-driven Current Operating Procedure for BEST was published in 2024.^69^ Building on this, a dedicated working group within the Insp-ECT consortium has since been established to develop Standardised Operating Procedures through a prospective clinical trial (NCT07579962). A randomised comparison with conventional sclerotherapy has yet to be initiated.

### Nanosecond pulsed field ablation for benign thyroid nodules

Benign thyroid nodules causing compressive symptoms such as difficulty breathing, swallowing, and neck pain are conventionally managed by thyroidectomy, which leads to loss of thyroid function and lifelong hormone replacement.^75^ Thermal ablation techniques (RFA, laser, and MWA) are alternative treatment options in specialist centres. However, their use near the recurrent laryngeal nerve, carotid artery, and trachea requires high operator skill, and approximately 20% of patients need retreatment within one year due to incomplete ablation and nodule regrowth. The fibrotic scarring caused by thermal ablation also complicates any subsequent surgery.

Nanosecond pulsed field ablation (nsPFA) is an emerging non-thermal technology developed by Pulse Biosciences (Hayward, CA, USA) for ablation of soft tissue in percutaneous and intraoperative procedures, such as treatment of benign thyroid nodules. The system delivers bipolar nanosecondduration electric pulses that induce regulated cell death rather than thermal necrosis.^75^ Treated cells release danger-associated molecular patterns that recruit immune cells to phagocytose the ablated material, leaving no residual fibrotic mass.

The results of the first-in-human feasibility study were published in 2025.^75^ The study enrolled 30 patients across three cohorts, treated with the: ablate and resect, partial nodule treatment, and full nodule treatment. Results showed that nsPFA caused cell death without heat-related damage or scarring, indicating the ability to treat tissue immediately adjacent to critical neurovascular structures.

In the five patients who received full-nodule treatment with therapeutic intent, mean volume reductions were 71.1% at one month and 85.8% at 12 months. Symptom relief was reported as early as two weeks post-procedure, with no serious adverse events in any cohort. Notably, this rate of shrinkage exceeded that typically observed with RFA, as four large clinical trials demonstrated that six months were required to achieve comparable volume reduction.^75^ nsPFA has tremendous promise for certain applications but the lesion size is limited by the potential thermal damage from the higher applied fields.

In March 2024, Pulse Biosciences announced FDA 510(k) clearance for the CellFX nsPFA Percutaneous Electrode System for soft tissue ablation. An ongoing multicentre clinical trial (NCT07226804) is further characterising the safety and efficacy profile of nsPFA in a larger cohort.

### Electroporation-mediated gene delivery

Electroporation-mediated gene delivery, commonly termed Gene Electro-Transfer (GET), represents a distinct application of reversible electroporation in which pulsed electric fields transiently permeabilise cell membranes to facilitate intracellular delivery of nucleic acids (plasmid DNA, RNA, or CRISPR constructs) without the use of viral vectors.^4,76,77^

Skin and skeletal muscle are the primary target tissues owing to their accessibility, and GET has progressed to clinical investigation across a broad range of indications including cancer immunotherapy, DNA vaccination against infectious diseases, and treatment of heritable conditions.^5,78-81^ For transdermal delivery, the stratum corneum presents a substantial barrier to delivery of hydrophilic and high-molecular-weight molecules. Advances in electrode design and pulse protocols, including non-invasive surface applicators, heat-assisted delivery, and combined electrode-reservoir devices, have progressively improved delivery depth, efficiency, and tolerability.^82-84^

Electroporation-mediated gene delivery to the myocardium (i.e. cardioporation) has shown feasibility for cardiac regeneration applications following myocardial infarction. In a rat model of cardiac ischemia, GET of VEGF-B encoding plasmid DNA, delivered directly to the ischemic myocardium, produced the first direct histological evidence of cardiomyogenesis post-infarction.^85^ Subsequent work further optimised cardioporation parameters, including electrode configuration and plasmid vector size, to increase expression efficiency while reducing ventricular fibrillation risk and tissue damage.^86^

Another opportunity of GET lies in the in vivo production of monoclonal antibodies (mAbs). Conventional mAbs are well established across oncology, autoimmune disease, and infectious disease, but their costly manufacture and need for repeated administration constrain accessibil ity.^87^ In DNA-based antibody therapy, rather than administering the antibody protein, plasmid DNA encoding the mAb is instead delivered to a tissue, such as skeletal muscle, which then serves as an in-situ source of sustained expression. Preclinical studies in small and large animals have demonstrated feasibility across diverse targets^88-90^, and the approach has recently progressed to first-inhuman evaluation.^91^

### Barriers to wider clinical adoption

Despite almost three decades of clinical experience, EBTTs remain outside mainstream treatment guidelines for most indications and are inconsistently reimbursed across geographical jurisdictions. Three interrelated categories of barriers account for this gap. First, the evidence base consists predominantly of single-arm series and registries, which are insufficient to drive guideline change or secure reimbursement decisions; the randomised comparative trials that regulators and health technology assessment bodies require have, with few exceptions, not been conducted. Second, the absence of validated treatment planning tools and consensus-based operating procedures limits reproducibility across centres and creates a practical expertise barrier that restricts dissemination beyond specialised institutions. Third, the regulatory and reimbursement landscape, particularly in Europe, is deeply fragmented, imposing a disproportionate burden on technologies addressing heterogeneous or rare indications.

### Randomised evidence and guideline integration

The most significant barrier to wider adoption of EBTTs is the scarcity of high-quality comparative evidence. After almost 30 years of promising single-arm data, oncological EBTTs remain on the margins of mainstream clinical practice, primarily because randomised trials were not conducted early enough. Registries, however valuable for safety surveillance and hypothesis generation, are insufficient to drive guideline changes or reimbursement decisions.

The challenge is compounded by the heterogeneity of oncological indications. For example, cardiac PFA benefits from an anatomically consistent target, the same catheter and technique apply across the patient population, whereas ECT or IRE ablation address tumours of various histology and sites, requiring different electrodes, different specialists, and different procedural approaches. A single pivotal trial is therefore impractical for oncological EBTTs. A pragmatic alternative is to design disease-specific trials that compare focal ablation (allowing centres to choose their preferred energy) against standard of care and then dissect the contributions of individual modalities in posthoc analyses. For rare cancers such as cholangio-carcinoma, multi-centre and potentially global collaboration is essential to accrue sufficient patient numbers.

The need for coordination of electroporation research across Europe was recognised more than a decade ago. COST Action TD1104 (2012–2016), the European Network for Development of Electroporation-Based Technologies and Treatments, united 73 researchers from 25 countries with the aims of streamlining European research and development activities, integrating multidisciplinary teams, providing comprehensive training for early-stage researchers, disseminating existing clinical applications, developing new applications, and standardising clinical protocols.^92^

**FIGURE 4.**
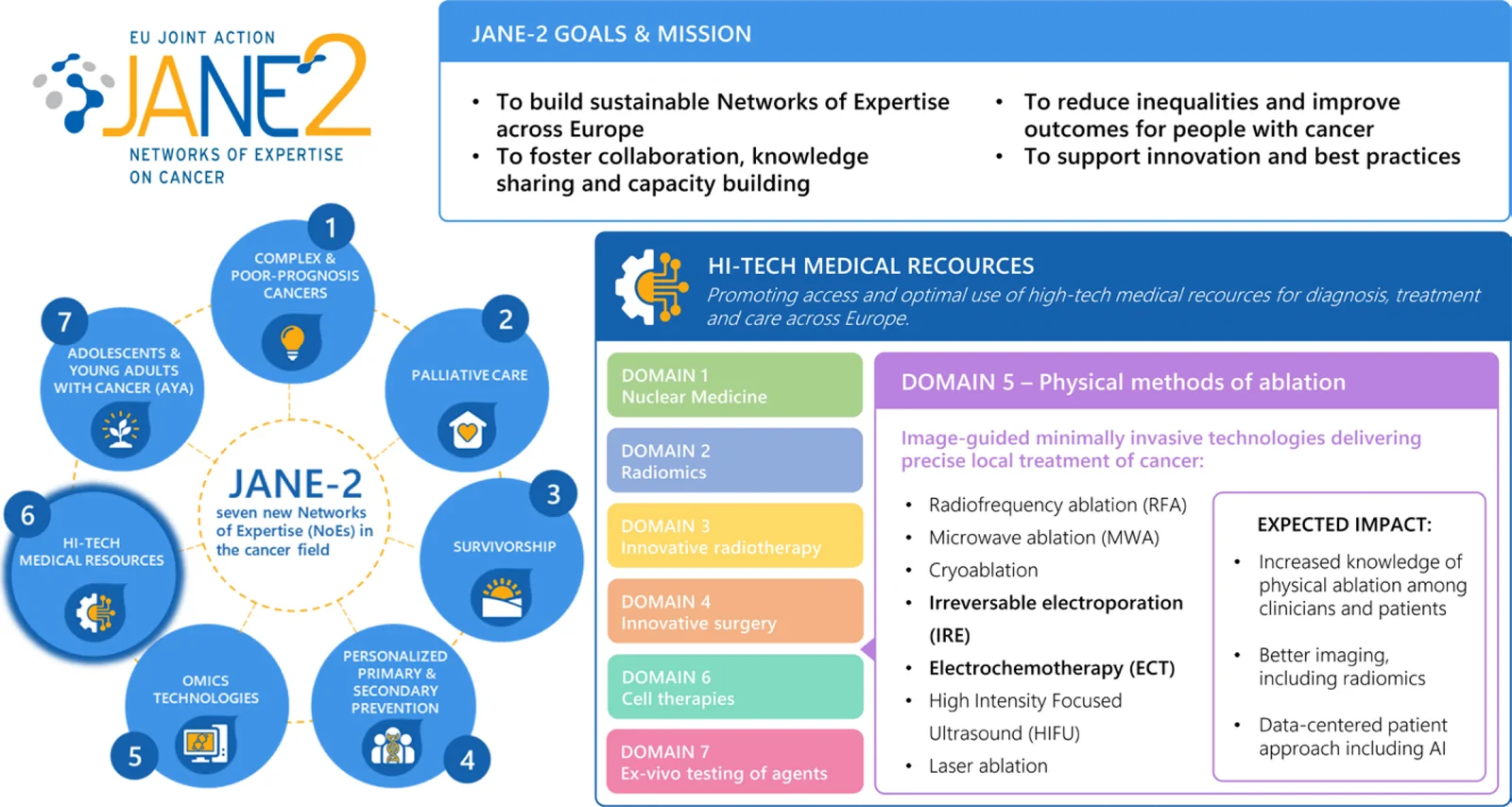
Overview of the JANE-2 (Joint Action on Networks of Expertise on Cancer) framework, highlighting WP10 – High-Tech Medical Resources and Domain 5 - Physical Methods of Ablation. The figure illustrates the position of Domain 5 within the broader network of JANE-2 and emphasizes its expected impact on advancing equitable access to innovative cancer treatment technologies across Europe.

Integration into Multidisciplinary Tumour Boards (MTBs) represents another critical gap. MTBs are the centrepiece of modern cancer care, yet EBTT expertise is not systematically represented; oncologists unfamiliar with ECT or IRE do not refer patients, and the technologies remain invisible at the point where treatment decisions are made. The EU Joint Action on Networks of Expertise in Cancer (JANE; https://jane-project.eu/) directly addresses this structural problem. Launched in 2022 under Europe’s Beating Cancer Plan, JANE has since expanded into JANE-2 (Figure 4) (https://jane-2.eu/), now encompassing 133 organisations from 29 EU countries and co-financed by the EU with €40.5 million.^93^

JANE is establishing Networks of Expertise across EU countries, with physical methods of ablation designated as one of seven priority medical resources (Figure 4). The initiative explicitly targets access inequities: patients across EU countries have widely unequal access to specialised ablation centres, and the absence of EBTTs from diseasespecific guidelines compounds this disparity. By embedding EBTT expertise within a formal network structure, JANE-2 creates the institutional conditions under which guideline inclusion, structured real-world data collection, and harmonised standard operating procedures become feasible at a European scale.^94^

### Treatment planning and monitoring

Adequate treatment planning remains an unresolved operational barrier for deep-seated EBTT targets. For cutaneous and subcutaneous ECT, fixed-geometry applicators and well-established standard operating procedures, most recently updated in 2018^20^, make the treatment zone predictable and the procedure reproducible across centres. No equivalent SOPs exist for ECT or IRE ablation of deep-seated tumours. Physicians performing these procedures must adhere or rely on guidelines developed for superficial targets, on institutional custom, or on the simplified 2D planning tools integrated into commercial electroporators (e.g., IGEA’s Cliniporator and Angiodynamics’ NanoKnifeÒ), which assume homogeneous tissue and perfectly parallel electrode placement, conditions that are rarely met in practice. In reality, tissue heterogeneity renders electric field distribution difficult to predict and anatomical constraints cause needle deflection.^27,95-97^

**FIGURE 5.**
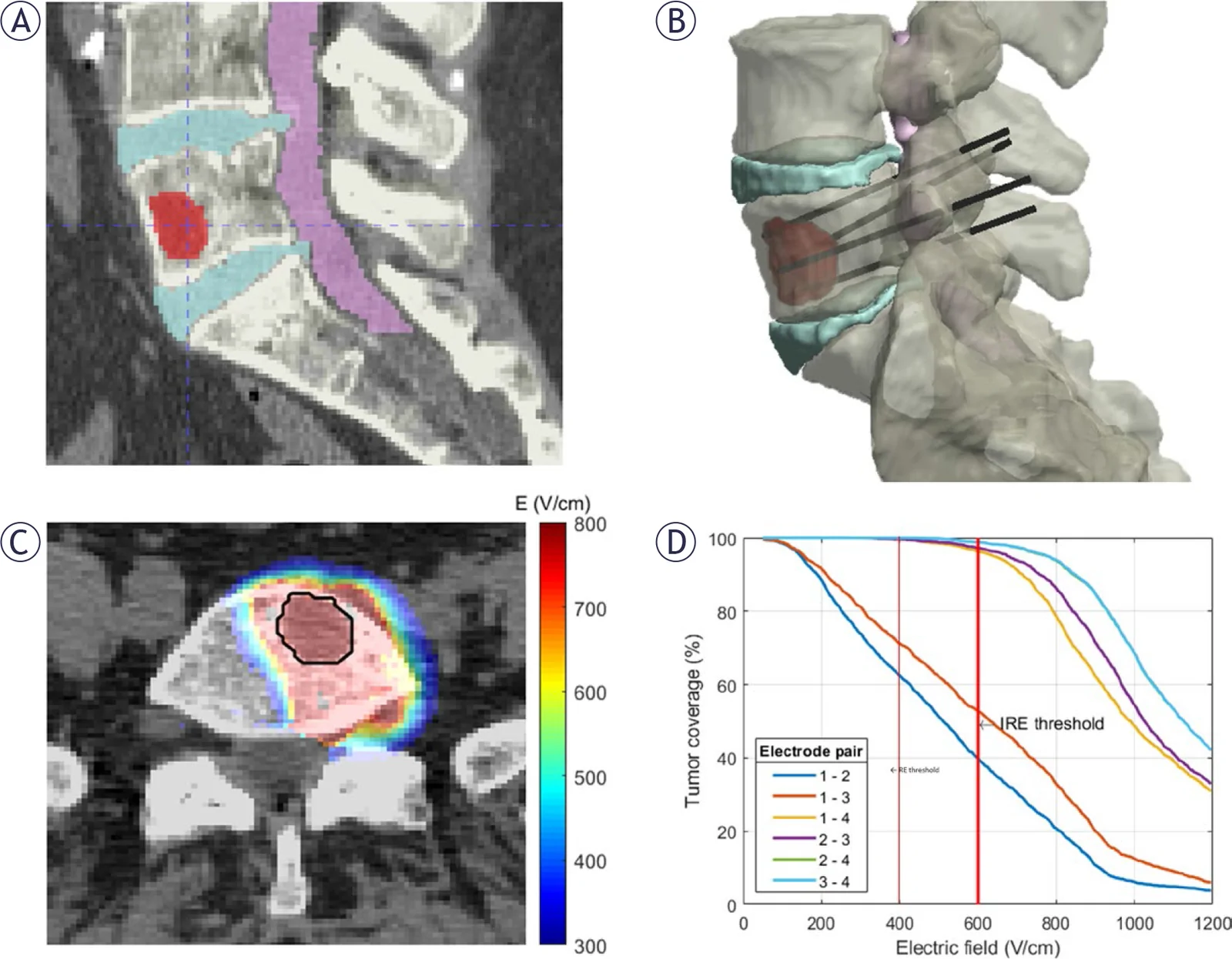
Illustration of a computer-assisted patient-specific treatment plan for electrochemotherapy of a spinal metastasis. **(A)** Segmentation of the tissues of interest (vertebrae, intervertebral discs, spinal cord, tumour) from the patient’s CT image. **(B)** Patient-specific 3D model developed from image segmentation showing the electrode placement for the selected treatment plan. **(C)** Predicted electric field distribution for the selected treatment plan, overlaid on the CT image (tumour outlined in black). **(D)** Cumulative tumour coverage as a function of electric field for the candidate electrode pairs; vertical lines mark the reversible (RE) and irreversible (IRE) electroporation thresholds. Adapted from.^106^

The clinical consequences of inadequate treatment planning are well illustrated in spinal ECT, where a 7.5% paraplegia rate has been attributed to uncontrolled current distribution near the spinal cord.^33^ Patient-specific 3D numerical models, constructed from CT or MRI data, can optimise electrode positioning and pulse parameters to achieve complete target coverage while minimising thermal injury and damage to sensitive structures (Figure 5).^98-101^ However, no treatment planning software for EBTTs is currently approved for clinical use, and prospective validation is lacking.

Furthermore, real-time and post-procedural assessment of the ablated zone constitutes a related unmet need. Unlike thermal ablation, where the necrotic zone is clearly demarcated on imaging and stable over time, the ablation zone after IRE undergoes rapid dynamic changes: it may transiently overestimate the true treatment volume in the first 24 hours, as both reversibly and irreversibly electroporated tissue can accumulate contrast agent, before shrinking considerably over the following weeks.^101-103^ The zone is also not internally uniform: histopathologic-radiologic correlation has shown that it comprises concentric layers of coagulative necrosis, congestion, and peripheral inflammation, each with distinct enhancement behaviour, such that different contrast phases over-or underestimate the true extent of cell death.^104,105^ Together, these dynamic changes make it difficult to determine with certainty the region of actual irreversible cell death on follow-up imaging, and there is currently no consensus on optimal posttreatment imaging timing or protocol.

Addressing these gaps in treatment planning and monitoring will require investment on several fronts: prospective clinical validation of treatment planning models, development of reliable intraprocedural monitoring, and integration of real-time planning with navigation systems. A further requirement is regulatory clearance for treatment planning software as a clinical-grade medical device. This is a demanding process that neither device manufacturers nor independent developers have so far pursued, leaving available tools confined to research use.^107-111^

### Regulatory pathways, reimbursement, and market fragmentation

The regulatory landscape for EBTT devices, while navigable, imposes significant time and cost burdens that disproportionately affect emerging applications and rare disease indications. EU Medical Device Regulation (MDR) certification through a Notified Body takes an average of 14 months (range 6 months to over 2 years), and each new clinical indication requires a separate body of clinical evidence, an obstacle for technologies that potentially address several different tumour types but cannot afford a clinical study for each. In the United States, the mean cost of bringing a novel Class III device to market has been estimated at approximately $54 million, with the pivotal clinical trial alone averaging around $31 million.^112^

In addition, reimbursement across Europe is deeply fragmented. Paradoxically, France, where ECT was pioneered, remains one of the most restrictive markets for the technology. Each country requires its own evidence package, business case, and often local clinical data. Clinical adoption in one jurisdiction does not automatically transfer to another, and evidence generated in one EU member country may not satisfy the reimbursement authorities of another. This fragmentation stands in contrast to the single FDA national approval pathway in the United States and constitutes a significant barrier for small and medium sized companies developing EBTT devices.

The cardiac PFA market illustrates how sufficient investments can overcome these barriers: companies routinely raise successive financing rounds in the tens to hundreds of millions of dollars, supported by the enormous global atrial fibrillation patient pool. Oncological and non-on-cological EBTT populations are smaller and more heterogeneous, requiring creative approaches to clinical trial design (adaptive, basket, or platform trials) that can generate sufficient evidence across multiple indications without requiring prohibitive investment for each.

## Conclusions and recommendations

Electroporation-based treatments and therapies represent a promising but underutilised family of clinical modalities. They can provide treatment opportunities for diseases that have not been adequately addressed so far and can improve patients’ quality of life (e.g. in patients with vascular malformations or with COPD) and offer disruptive treatments (e.g. for type 2 diabetes and cardiac arrhythmia treatment). The explosive growth of cardiac PFA has renewed interest in this platform technology and demonstrated that electroporation can achieve mainstream clinical adoption when the necessary conditions are met. The emerging applications reviewed here confirm that the technology’s translational potential extends well beyond its traditional oncological and cardiac domains. Based on the collective expert opinion of the authors, we offer the following recommendations to facilitate wider clinical adoption of EBTTs.

1. **Pursue targeted randomised trials**. Comparative evidence is the main barrier to guideline inclusion. Trials should be prioritised for indications where randomisation is ethical and feasible, such as ECT versus palliative radiotherapy for spinal metastases. Designs comparing focal ablation with standard care can generate actionable evidence.
2. **Develop indication-specific standard operating**. procedures The ECT SOP model has been instrumental in enabling reproducible results across centres. Equivalent consensus-based SOPs are needed for IRE of deep-seated tumours, ECT of spinal metastases, and the emerging non-oncological applications; a recent example is the publication of Current Operating Procedures for BEST with a prospective trial currently underway to evaluate it in clinical practice.
3. **Invest in treatment planning infrastructure**. Reliable patient-specific treatment planning remains a critical gap for deep-seated tumour ablation and complex non-oncological procedures. Validated, regulatory-compliant software is urgently needed, and academic groups should collaborate with device manufacturers to pursue bundled device-software submissions that can streamline the regulatory pathway for both.
4. **Embed EBTTs in multidisciplinary decisionmaking**. Electroporation expertise must be systematically represented in multidisciplinary oncology boards and, for non-oncological applications, in the relevant disease-specific clinical forums. The EU JANE initiative provides a ready framework for embedding this expertise at the European level.
5. **Harmonise European reimbursement pathways**. The current country-by-country fragmentation of reimbursement criteria discourages investment and limits patient access. Europeanlevel harmonisation of evidence requirements for physical ablation modalities would substantially lower the adoption barrier for both established and emerging EBTT applications.

The tools exist, the evidence of biological efficacy is compelling, and the clinical need is clear. The task now is institutional: building the evidentiary, regulatory, and organisational infrastructure to deliver electroporation-based treatments to patients who stand to benefit most.

## References

[1] Kotnik T, Rems L, Tarek M, Miklavčič D. (2019). Membrane electroporation and electropermeabilization: mechanisms and models. Annu Rev Biophys.

[2] Serša G, Miklavcic D, Cemazar M, Rudolf Z, Pucihar G, Snoj M. (2008). Electrochemotherapy in treatment of tumours. Eur J. Surg Oncol.

[3] Orlowski S, Belehradek J, Paoletti C, Mir LM. (1988). Transient electropermeabilization of cells in culture: increase of the cytotoxicity of anticancer drugs. Biochem Pharmacol.

[4] Heller R, Heller LC., Huang L, Liu D, Wagner E (2015). Advances in Genetics.

[5] Sachdev S, Potočnik T, Rems L, Miklavčič D. (2022). Revisiting the role of pulsed electric fields in overcoming the barriers to *in vivo* gene electrotransfer. Bioelectrochemistry.

[6] Davalos RV, Mir ILM, Rubinsky B. (2005). Tissue ablation with irreversible electroporation. Ann Biomed Eng.

[7] Geboers B, Scheffer HJ, Graybill PM, Ruarus AH, Nieuwenhuizen S, Puijk RS (2020). High-voltage electrical pulses in oncology: irreversible electroporation, electrochemotherapy, gene electrotransfer, electrofusion, and electroimmunotherapy. Radiology.

[8] Ekanem E, Neuzil P, Reichlin T, Kautzner J, Van Der Voort P, Jais P (2024). Safety of pulsed field ablation in more than 17,000 patients with atrial fibrillation in the MANIFEST-17K study. Nat Med.

[9] Miklavčič D, Verma A, Krahn PRP, Štublar J, Kos B, Escartin T (2024). Biophysics and electrophysiology of pulsed field ablation in normal and infarcted porcine cardiac ventricular tissue. Sci Rep.

[10] Cannon R, Ellis S, Hayes D, Narayanan G, Martin RCG. (2013). Safety and early efficacy of irreversible electroporation for hepatic tumors in proximity to vital structures. J Surg Oncol.

[11] Sutter O, Calvo J, N’Kontchou G, Nault J-C, Ourabia R, Nahon P (2017). Safety and efficacy of irreversible electroporation for the treatment of hepatocellular carcinoma not amenable to thermal ablation techniques: a retrospective single-center case series. Radiology.

[12] Jarm T, Cemazar M, Miklavcic D, Sersa G. (2010). Antivascular effects of electrochemotherapy: implications in treatment of bleeding metastases. Expert Rev Anticancer Ther.

[13] Brinton M, Mandel Y, Schachar I, Palanker D. (2018). Mechanisms of electrical vasoconstriction. J Neuroeng Rehabil.

[14] Calvet CY, Mir LM. (2016). The promising alliance of anti-cancer electrochemotherapy with immunotherapy. Cancer Metastasis Rev.

[15] Scheffer HJ, Stam AGM, Geboers B, Vroomen LGPH, Ruarus A, de Bruijn B (2019). Irreversible electroporation of locally advanced pancreatic cancer transiently alleviates immune suppression and creates a window for antitumor T cell activation. Oncoimmunology.

[16] Zhao J, Wen X, Tian L, Li T, Xu C, Wen X (2019). Irreversible electroporation reverses resistance to immune checkpoint blockade in pancreatic cancer. Nat Commun.

[17] Kesar U, Markelc B, Jesenko T, Valentinuzzi KU, Cemazar M, Strojan P (2023). Effects of electrochemotherapy on immunologically important modifications in tumor cells. Vaccines.

[18] Mir LM, Belehradek M, Domenge C, Orlowski S, Poddevin B, Belehradek J (1991). [Electrochemotherapy, a new antitumor treatment: first clinical trial]. [French]. C R AcadSci III.

[19] Mir LM, Gehl J, Sersa G, Collins CG, Garbay J-R, Billard V (2006). Standard operating procedures of the electrochemotherapy: instructions for the use of bleomycin or cisplatin administered either systemically or locally and electric pulses delivered by the CliniporatorTM by means of invasive or non-invasive electrodes. Eur J Cancer Suppl.

[20] Gehl J, Sersa G, Matthiessen LW, Muir T, Soden D, Occhini A (2018). Updated standard operating procedures for electrochemotherapy of cutaneous tumours and skin metastases. Acta Oncol.

[21] Campana LG, Edhemovic I, Soden D, Perrone AM, Scarpa M, Campanacci L (2019). Electrochemotherapy - emerging applications technical advances, new indications, combined approaches, and multi-institutional collaboration. Eur J Surg Oncol.

[22] Clover AJP, de Terlizzi F, Bertino G, Curatolo P, Odili J, Campana LG (2020). Electrochemotherapy in the treatment of cutaneous malignancy: outcomes and subgroup analysis from the cumulative results from the pan-European International Network for Sharing Practice in Electrochemotherapy database for 2482 lesions in 987 patients (2008–2019). Eur J Cancer.

[23] Lyons P, Polini D, Russell-Ryan K, Clover AJP. (2023). High-frequency electroporation and chemotherapy for the treatment of cutaneous malignancies: evaluation of early clinical response. Cancers (Basel).

[24] Linnert M, Iversen HK, Gehl J. (2012). Multiple brain metastases - current management and perspectives for treatment with electrochemotherapy. Radiol Oncol.

[25] Djokic M, Cemazar M, Popovic P, Kos B, Dezman R, Bosnjak M (2018). Electrochemotherapy as treatment option for hepatocellular carcinoma, a prospective pilot study. Eur J Surg Oncol.

[26] Izzo F, Granata V, Fusco R, D’Alessio V, Petrillo A, Lastoria S (2021). Clinical Phase I/II study: local disease control and survival in locally advanced pancreatic cancer treated with electrochemotherapy. J Clin Med.

[27] Luerken L, Doppler M, Brunner SM, Schlitt HJ, Uller W. (2021). Stereotactic percutaneous electrochemotherapy as primary approach for unresectable large HCC at the hepatic hilum. Cardiovasc Intervent Radiol.

[28] Campanacci L, Cevolani L, De Terlizzi F, Saenz L, Alì N, Bianchi G (2022). Electrochemotherapy is effective in the treatment of bone metastases. Curr Oncol.

[29] Granata V, Fusco R, D’Alessio V, Simonetti I, Grassi F, Silvestro L (2023). Percutanous electrochemotherapy (ECT) in primary and secondary liver malignancies: a systematic review. Diagnostics.

[30] Stevanovic M, Heringer M, Hjouj M, Zanasi A, Terlizzi F de, Stehling MK. (2025). Prostate cancer treatment with electrochemotherapy (ECT): safety, efficacy and clinical experience in 144 patients. Radiol Oncol.

[31] Vivod G, Cilensek I, Kovacevic N, Sersa G, Cemazar M, Merlo S. (2025). Quality of life of women with recurrent vulvar cancer treated with electrochemotherapy. Radiol Oncol.

[32] Deschamps F, Tselikas L, Yevich S, Bonnet B, Roux C, Kobe A (2023). Electrochemotherapy in radiotherapy-resistant epidural spinal cord compression in metastatic cancer patients. Eur J Cancer.

[33] Deschamps F, Tselikas L, Cazzato RL, Facchini G, Granata V, Bonnet B (2025). Electrochemotherapy in metastatic epidural spinal cord compression: a review and technical update. Br J Radiol.

[34] Falk Hansen H, Bourke M, Stigaard T, Clover J, Buckley M, O’Riordain M (2020). Electrochemotherapy for colorectal cancer using endoscopic electroporation: a phase 1 clinical study. Endosc Int Open.

[35] Broholm M, Vogelsang R, Bulut M, Gögenur M, Stigaard T, Orhan A (2024). Neoadjuvant calcium electroporation for potentially curable colorectal cancer. Surg Endosc.

[36] Adeyeye A, Haji A. (2025). Establishing a salvage endoscopic electroporation (SEE) service for colorectal cancer: the King’s protocol for clinical implementation. J Clin Med.

[37] Adeyeye A, Olabintan O, Ayubi H, Gao H, Saini A, Emmanuel A (2025). Palliative luminal treatment of colorectal cancer using endoscopic calcium-electroporation: first case series from United Kingdom. J Clin Med.

[38] Bonura GF, Gualandi N, Soriani P, Cortegoso Valdivia P, Gabbani T, Zadro V (2025). Pioneering endoscopic calcium-electroporation in gastric cancer: a case series of an emerging therapeutic approach. Diseases.

[39] Pellegrino R, Nacca V, Paragliola F, Martinelli E, Federico A, Gravina AG. (2026). Endoscopic calcium electroporation for unfit-for-surgery bleeding colorectal cancer: the dawn of a new treatment?. Minerva Med.

[40] Egeland C, Baeksgaard L, Johannesen HH, Löfgren J, Plaschke CC, Svendsen LB (2018). Endoscopic electrochemotherapy for esophageal cancer: a phase I clinical study. Endosc Int Open.

[41] Egeland C, Baeksgaard L, Gehl J, Gögenur I, Achiam MP. (2022). Palliative treatment of esophageal cancer using calcium electroporation. Cancers (Basel).

[42] Cvetkoska A, Pírc E, Reberšek M, Magjarević R, Míklavčič D. (2020). Towards standardization of electroporation devices and protocols. IEEE Instrum Meas Mag.

[43] Al-Sakere B, André F, Bernat C, Connault E, Opolon P, Davalos RV (2007). Tumor ablation with irreversible electroporation. PLoS One.

[44] Narayanan G, Gentile NT, Eyshi J, Schiro BJ, Gandhi RT, Peña CS (2024). Irreversible electroporation in treating colorectal liver metastases in proximity to critical structures. J Vasc Interv Radiol.

[45] van den Bos W, Scheltema MJ, Siriwardana AR, Kalsbeek AMF, Thompson JE, Ting F (2018). Focal irreversible electroporation as primary treatment for localized prostate cancer. BJU Int..

[46] Niessen C, Thumann S, Beyer L, Pregler B, Kramer J, Lang S (2017). Percutaneous irreversible electroporation: Long-term survival analysis of 71 patients with inoperable malignant hepatic tumors. Sci Rep.

[47] Martin RCG, Durham AN, Besselink MG, Iannitti D, Weiss MJ, Wolfgang CL (2016). Irreversible electroporation in locally advanced pancreatic cancer: a call for standardization of energy delivery. J Surg Oncol.

[48] Narayanan G, Hosein PJ, Beulaygue IC, Froud T, Scheffer HJ, Venkat SR (2017). Percutaneous image-guided irreversible electroporation for the treatment of unresectable, locally advanced pancreatic adenocarcinoma. J Vasc Interv Radiol.

[49] Ruarus AH, Vroomen LGPH, Geboers B, van Veldhuisen E, Puijk RS, Nieuwenhuizen S (2019). Percutaneous irreversible electroporation in locally advanced and recurrent pancreatic cancer (PANFIRE-2): a multicenter, prospective, single-arm, phase II study. Radiology.

[50] Reddy VY, Koruth J, Jais P, Petru J, Timko F, Skalsky I (2018). Ablation of atrial fibrillation with pulsed electric fields: an ultra-rapid, tissue-selective modality for cardiac ablation. JACC Clin Electrophysiol.

[51] Verma A, Haines DE, Boersma LV, Sood N, Natale A, Marchlinski FE (2023). Pulsed field ablation for the treatment of atrial fibrillation: PULSED AF pivotal trial. Circulation.

[52] Della Rocca DG, Cespón-Fernández M, Keelani A, Raffa S, Pannone L, Almorad A (2024). Focal pulsed field ablation for premature ventricular contractions: a multicenter experience. Circ Arrhythm Electrophysiol.

[53] Reddy VY, Gerstenfeld EP, Natale A, Whang W, Cuoco FA, Patel C (2023). Pulsed field or conventional thermal ablation for paroxysmal atrial fibrillation. N Engl J Med.

[54] Reichlin T, Kueffer T, Badertscher P, Jüni P, Knecht S, Thalmann G (2025). Pulsed field or cryoballoon ablation for paroxysmal atrial fibrillation. N Engl J Med.

[55] Jais P, Neuzil P, Scherr D, Frison E, Knecht S, Boveda S (2026). Pulsed field vs radiofrequency ablation for paroxysmal atrial fibrillation: the BEAT PAROXAF trial. Eur Heart J.

[56] Reddy VY, Gerstenfeld EP, Schmidt B, Nair D, Natale A, Saliba W (2025). Pulsed field ablation for persistent atrial fibrillation: 1-Year Results of ADVANTAGE AF. J Am Coll Cardiol.

[57] Kühne M, Badertscher P, Andrade JG, Anic A, Chun J, Dello Russo A (2026). Pulsed field ablation for the interventional treatment of atrial fibrillation. A scientific statement of the European Heart Rhythm Association (EHRA) of the ESC, the Heart Rhythm Society (HRS), the Asia Pacific Heart Rhythm Society (APHRS), the Latin American Heart Rhythm Society (LAHRS) and the Canadian Heart Rhythm Society (CHRS). Europace.

[58] Verma A, Hocini M, Andrade J, Gerstenfeld E, Kapa S, Lakkireddy D (2026). 2026 HRS/EHRA scientific statement on pulsed field ablation for cardiac arrhythmias. Heart Rhythm.

[59] Dasa O, Younis A, Higuchi K, Sroubek J, Lee JZ, Chung R (2026). Early U.S. experience with catheter ablation of ventricular arrhythmias with a dual-energy lattice-tip catheter: insights from the CLEAR-VT registry. JACC Clin Electrophysiol.

[60] Busch CBE, Meiring S, van Baar ACG, Holleman F, Nieuwdorp M, Bergman JJGHM. (2024). Recellularization via electroporation therapy of the duodenum combined with glucagon-like peptide-1 receptor agonist to replace insulin therapy in patients with type 2 diabetes: 12-month results of a first-inhuman study. Gastrointest Endosc.

[61] O’Neal DN, Sartoretto A, Holt BA, Abu Dayyeh BK, Vaughan R, Cameron G (2026). Safety, feasibility, and dose-dependent metabolic effects of endoscopic duodenal pulsed electric field therapy in adults with type 2 diabetes: results from two multicentre proof-of-concept studies. Diabetes Obes Metab.

[62] Abu Dayyeh BK, Asirvatham SJ. (2024). Nonthermal pulsed electric field recellularization in the duodenum for type 2 diabetes mellitus. VideoGIE.

[63] Valipour A, Fernandez-Bussy S, Ing AJ, Steinfort DP, Snell GI, Williamson JP (2020). Bronchial rheoplasty for treatment of chronic bronchitis. Twelvemonth results from a multicenter clinical trial. Am J Respir Crit Care Med.

[64] Sciurba FC, Dransfield MT, Kim V, Marchetti N, Comellas A, Hogarth DK (2023). Bronchial rheoplasty for chronic bronchitis: 2-year results from a US feasibility study with RheOx. BMJ Open Respir Res.

[65] Brock J, Herth F, Darwiche K, Hübner R-H, Skowasch D, Schwick B (2025). Bronchial rheoplasty for chronic bronchitis: real-world evidence of safety and performance. ERJ Open Res.

[66] Meininger GR, Neal RE, Hunter DW, Krimsky WS. (2024). Absence of arrhythmogenicity with biphasic pulsed electric fields delivered to porcine airways. Ann Biomed Eng.

[67] Muir T, Bertino G, Groselj A, Ratnam L, Kis E, Odili J (2023). Bleomycin electrosclerotherapy (BEST) for the treatment of vascular malformations. An International Network for Sharing Practices on Electrochemotherapy (InspECT) study group report. Radiol Oncol.

[68] Haehl J, Haeberle B, Muensterer O, Hartel A, Fröba-Pohl A, Dussa CU (2025). Bleomycin electrosclerotherapy (BEST) of slow-flow vascular malformations (SFVMs) in children. J Pediatr Surg.

[69] Muir T, Wohlgemuth WA, Cemazar M, Bertino G, Groselj A, Ratnam LA (2024). Current Operating Procedure (COP) for Bleomycin ElectroScleroTherapy (BEST) of low-flow vascular malformations. Radiol Oncol.

[70] Sersa G, Krzic M, Sentjurc M, Ivanusa T, Beravs K, Kotnik V (2002). Reduced blood flow and oxygenation in SA-1 tumours after electrochemotherapy with cisplatin. Br J Cancer.

[71] Lisec B, Cemazar M, Muir T, Omerzel M, Jesenko T, Markelc B (2026). Bleomycin ElectroScleroTherapy (BEST): mechanistic parallels to electrochemotherapy, experimental models, and unresolved questions. Radiol Oncol.

[72] Wohlgemuth WA, Müller-Wille R, Meyer L, Wildgruber M, Guntau M, Heydt S von der (2021). Bleomycin electrosclerotherapy in therapy-resistant venous malformations of the body. J Vasc Surg Venous Lymphat Disord.

[73] Schmidt VF, Cangir Ö, Meyer L, Goldann C, Hengst S, Brill R (2024). Outcome of bleomycin electrosclerotherapy of slow-flow malformations in adults and children. Eur Radiol.

[74] Loeser JH, Guntau M, Bidakov O, von der Heydt S, Gussew A, Schob S (2025). Therapy of lymphatic malformations or isolated lymphatic components in combined slow-flow malformations with bleomycin electrosclerotherapy (BEST). Rofo.

[75] Spiezia S, Offi C, Misso C, Antonelli G, Nuccitelli R, Tufano RP (2025). First-in-human clinical feasibility study of ablation of benign thyroid nodules using nanosecond pulsed field ablation. Thyroid.

[76] Lambricht L, Lopes A, Kos S, Sersa G, Préat V, Vandermeulen G. (2016). Clinical potential of electroporation for gene therapy and DNA vaccine delivery. Expert Opin Drug Deliv.

[77] Rosazza C, Meglic SH, Zumbusch A, Rols M-P, Miklavcic D. (2016). Gene Electrotransfer: a Mechanistic Perspective. Curr Gene Ther.

[78] Heller LC, Singh JS, Synowiec JC, Cherukuri PK, Phan N, Shi G (2025). Effective gene immunotherapy for melanoma utilizing an advanced *in vivo* electrotransfer system. Mol Ther Oncol.

[79] Strojan P, Jesenko T, Omerzel M, Jamsek C, Groselj A, Tratar UL (2025). Phase I trial of phIL12 plasmid intratumoral gene electrotransfer in patients with basal cell carcinoma in head and neck region. Eur J Surg Oncol.

[80] Daud AI, DeConti RC, Andrews S, Urbas P, Riker AI, Sondak VK (2008). Phase I trial of interleukin-12 plasmid electroporation in patients with metastatic melanoma. J Clin Oncol.

[81] Spanggaard I, Snoj M, Cavalcanti A, Bouquet C, Sersa G, Robert C (2013). Gene electrotransfer of plasmid antiangiogenic metargidin peptide (AMEP) in disseminated melanoma: safety and efficacy results of a phase I first-in-man study. Hum Gene Ther Clin Dev.

[82] Bulysheva A, Heller L, Francis M, Varghese F, Boye C, Heller R. (2021). Monopolar gene electrotransfer enhances plasmid DNA delivery to skin. Bioelectrochemistry.

[83] Edelblute C, Mangiamele C, Heller R. (2021). Moderate heat-assisted gene electrotransfer as a potential delivery approach for protein replacement therapy through the skin. Pharmaceutics.

[84] Simon J, Jouanmiqueou B, Rols M-P, Flahaut E, Golzio M. (2021). Transdermal delivery of macromolecules using two-in-one nanocomposite device for skin electroporation. Pharmaceutics.

[85] Bulysheva AA, Burcus N, Lundberg CG, Francis MP, Heller R. (2018). VEGF-B electrotransfer mediated gene therapy induces cardiomyogenesis in a rat model of cardiac ischemia. Bioelectrochemistry.

[86] Boye C, Arpag S, Burcus N, Lundberg C, DeClemente S, Heller R (2021). Cardioporation enhances myocardial gene expression in rat heart. Bioelectrochemistry.

[87] Aboul-Ella H, Gohar A, Ali AA, Ismail LM, Mahmoud AEE-R, Elkhatib WF (2024). Monoclonal antibodies: from magic bullet to precision weapon. Mol Biomed.

[88] Hollevoet K, De Smidt E, Geukens N, Declerck P. (2018). Prolonged in vivo expression and anti-tumor response of DNA-based anti-HER2 antibodies. Oncotarget.

[89] Esquivel RN, Patel A, Kudchodkar SB, Park DH, Stettler K, Beltramello M (2019). In vivo delivery of a DNA-encoded monoclonal antibody protects non-human primates against Zika virus. Mol Ther.

[90] Cuypers M-L, Geukens N, Hollevoet K, Declerck P, Dewilde M. (2023). Exploring the fate of antibody-encoding pDNA after intramuscular electroporation in mice. Pharmaceutics.

[91] Tebas P, Patel A, Agnes JT, Parzych EM, Baer A, Caturla M (2025). Safety and pharmacokinetics of SARS-CoV-2 DNA-encoded monoclonal antibodies in healthy adults: a phase 1 trial. Nat Med.

[92] Miklavčič D. (2012). Network for development of electroporation-based technologies and treatments: COST TD1104. J Membr Biol.

[93] Gehl J, Pereira PL, Cantwell CP, Deschamps F, Kocijancic A, Schmidt N (2026). Tumor ablation: emerging uses, challenges, and strategic implementation. A green paper by the Network of Expertise in Cancer (JANE-2), High Tech Medical Resources, network on Physical Methods of Tumor Ablation. Radiol Oncol.

[94] Casali PG, Antoine-Poirel H, Berrocoso S, Blay J-Y, Dubois T, Ferrari A (2025). Health networking on cancer in the European Union: a ‘green paper’ by the EU Joint Action on Networks of Expertise (JANE). ESMO Open.

[95] Mathy RM, Tinoush P, da Florencia RD, Braun A, Ghamarnejad O, Radeleff B (2020). Impact of needle positioning on ablation success of irreversible electroporation: a unicentric retrospective analysis. Sci Rep.

[96] Cindrič H, Kos B, Miklavčič D., Impellizeri JA (2021). Electroporation in veterinary oncology practice: electrochemotherapy and gene electrotransfer for immunotherapy.

[97] Andrade DLLS, Guedert R, Pintarelli GB, Rangel MMM, Oliveira KD, Quadros PG (2022). Electrochemotherapy treatment safety under parallel needle deflection. Sci Rep.

[98] Grošelj A, Kos B, Čemažar M, Urbančič J, Kragelj G, Bošnjak M (2015). Coupling treatment planning with navigation system: a new technological approach in treatment of head and neck tumors by electrochemotherapy. Biomed Eng Online.

[99] Kos B, Voigt P, Miklavcic D, Moche M. (2015). Careful treatment planning enables safe ablation of liver tumors adjacent to major blood vessels by percutaneous irreversible electroporation (IRE). Radiol Oncol.

[100] Garcia PA, Kos B, Rossmeisl JH, Pavliha D, Miklavčič D, Davalos RV. (2017). Predictive therapeutic planning for irreversible electroporation treatment of spontaneous malignant glioma. Med Phys.

[101] Cindrič H, Mariappan P, Beyer L, Wiggermann P, Moche M, Miklavčič D (2021). Retrospective study for validation and improvement of numerical treatment planning of irreversible electroporation ablation for treatment of liver tumors. IEEE Trans Biomed Eng.

[102] Padia SA, Johnson GE, Yeung RS, Park JO, Hippe DS, Kogut MJ. (2016). Irreversible electroporation in patients with hepatocellular carcinoma: immediate versus delayed findings at MR imaging. Radiology.

[103] Barabasch A, Distelmaier M, Heil P, Krämer NA, Kuhl CK, Bruners P. (2017). Magnetic resonance imaging findings after percutaneous irreversible electroporation of liver metastases: a systematic longitudinal study. Invest Radiol.

[104] Felker ER, Dregely I, Chung DJ, Sung K, Osuagwu FC, Lassman C (2017). Irreversible electroporation: defining the MRI appearance of the ablation zone with histopathologic correlation in a porcine liver model. AJR Am J Roentgenol.

[105] Granata V, Fusco R, Salati S, Petrillo A, Bernardo ED, Grassi R (2021). A Systematic review about imaging and histopathological findings for detecting and evaluating electroporation based treatments response. Int J Environ Res Public Health.

[106] Cindrič H, Kos B, Tedesco G, Cadossi M, Gasbarrini A, Miklavčič D. (2018). Electrochemotherapy of spinal metastases using transpedicular approach - a numerical feasibility study. Technol Cancer Res Treat.

[107] Pavliha D, Kos B, Marčan M, Zupanič A, Serša G, Miklavčič D. (2013). Planning of electroporation-based treatments using Web-based treatment-planning software. J Membr Biol.

[108] Marčan M, Pavliha D, Kos B, Forjanič T, Miklavčič D. (2015). Web-based tool for visualization of electric field distribution in deep-seated body structures and planning of electroporation-based treatments. Biomed Eng Online.

[109] Perera-Bel E, Yagüe C, Mercadal B, Ceresa M, Beitel-White N, Davalos RV (2020). EView: an electric field visualization web platform for electroporationbased therapies. Comput Methods Programs Biomed.

[110] Perera-Bel E, Aycock KN, Salameh ZS, Gómez-Barea M, Davalos RV, Ivorra A (2022). PIRET - a platform for treatment planning in electroporationbased therapies. IEEE Trans Biomed Eng.

[111] Paffi A, Apollonio F, Cadossi M, D’Alessio V, Fusco R, Giannini A (2024). A fast 3-D approach for electroporation treatment planning: optimal electrodes configuration. IEEE J Electromagn RF Microw Med Biol.

[112] Sertkaya A, DeVries R, Jessup A, Beleche T. (2022). Estimated cost of developing a therapeutic complex medical device in the US. JAMA Netw Open.

